# Clinical Lecture on Malignnt Stricture of the Hepatic Flexure of the Colon

**Published:** 1879-11

**Authors:** James E. Goodhart

**Affiliations:** Assistant Physician to Guy’s Hospital and the Evelina Hospital for Children


### Selections.

CLINICAL LECTURE ON MALIGNANT STRICTURE OF THE HEPATIC FLEXURE OF THE COLON. By JAMES E. GOODHART, M.D., Assistant Physician to Guy's Hospital and the Evelina Hospital for Children.

The case to which I invite your attention, gentlemen, to-day is one of stricture of the colon; but, in order that those of you who know little about such things may follow me the more readily, it will be advisable in the first place, to mention the common symptoms of intestinal obstruction, and also the varioas causes of the same.

The symptoms are, vomiting; griping abdominal pain; constipation; more or less distention of the abdomen; abnormally visible peristaltic action of the bowels; and possibly, in some cases, diminished quantity of urine.

According as the obstruction is sudden and complete, or slow and for a long time incomplete, in its onset, you can readily understand that the symptoms will be likely to differ. Thus, in sudden cases, the vomiting and constipation are immediate, and the abdominal distension follows soon after. In the chronic cases, there is usually a history of paroxysmal colic for some time before the onset of more urgent symptoms. The abdominal distension slowly increases, and reaches an extreme degree, and the vomiting and absolute constipation come on at this late period. Moreover, throughout the illness, the visible muscular action is usually a prominent symptom. This is, I think, the simplest division of intestinal obstruction—into acute and chronic; and you will find that the various forms are readily classified in this way for clinical interpretation. It is not very elaborate, and therefore proportionately liable to error for exceptional cases; but it is correct for the majority of cases. Some writers attempt to classify according to the seat of the disease; this comes very much to the same thing, but is not quite so simple.

The following causes are usually enumerated:

| | |
|---|---|
| | 1. Plugging of the intestine by foreign bodies and concretions. |
| Acute. | 2. Internal strangulation.<br>3. Volvulus. |
| Acute or Chronic. | 4. Intussusception. |
| Chronic. | 5. Contractions.<br>6. Strictures. |

Of these six, the first, except when due to a large gall-stone, is very rare, so that it need not detain us. Nos. 2 and 3 are associated with very acute symptoms, early vomiting, pain, and distension of the abdomen. Intussusception occupies a somewhat intermediate position, as it is sometimes acute, sometimes chronic; and 5 and 6 usually have chronic symptoms.

The case is that of a man aged 27, admitted into the clinical ward on May 22nd, 1878, and who died on May 31st. He was an Irishman, born in Killarney, and had had no illness that he was aware of, with the exception of measles, and, four years ago, of gonorrhœa. His father and mother are alive and healthy. He has drunk very freely of spirits and beer, being for some time a bottler of whisky, when he used to imbibe more than a pint *per diem*. From Killarney, he went to a sugar-factory at Bristol, where he used to drink considerably more than three quarts of beer in the day. During the last twelve months, he has not drunk nearly so much, only taking spirits at long intervals, and about a quart of beer per day. During the last nine months, the patient has suffered from attacks of griping pains in his right side, more especially after taking beer; he has never noticed that the pain was increased by food; and he has been so little troubled by it as to take not much notice of it.

About two months ago, he caught a bad cold, and was confined to his bed, and treated by a medical man. He then first noticed a hard swelling in his right side, just below the ribs. Previously, he had felt in good health, and had been able to work and eat well. He now began to have pain in this spot; and though he got better of his cold, and began to work again, he felt weak, and his appetite failed; a month ago he was seized with severe pain in his right side and for two days his bowels were confined. This attack passed off, but only to be succeeded by another in a week's time. The pains generally came on in the evening, and, after lasting twenty-four or thirty-six hours, would go away. During the pain, he was always sick, and, in so doing, was relieved. The vomit was bilious, green or yellow. Sickness never came on immediately after food, nor has he ever thrown up any blood, but the ejecta have been of the nature of coffee-grounds. The vomiting has only existed for the last four weeks. The bowels have been irregular, at one time constipated, at another loose. The motions have lately been very black. He thinks he has wasted in the last three weeks. He has been able to take solid food till the last week, but since then has kept to milk and brandy.

When admitted, he is described by my clerk, Mr. Malpas, as of a dark and sallow complexion, slightly jaundiced, with worn, pained, and somewhat pinched-looking face. He lies in bed on his back, with his legs drawn up; this being his most easy position. His tongue was furred and moist. He was always thirsty, but quite without appetite. The bowels had acted not long before he came in.

The abdomen was not at all distended—rather the reverse; so much so, that a tumor now to be described gave a slight prominence to the surface immediately over it. It occupied the right hypochondrium and right umbilical region, extending for an inch below the umbilicus. Its lower edge was well defined, but not its upper; the whole mass very tender and very hard. It was comparatively dull, but yet distinctly resonant on percussion, and between it and the right rib there was very distinct resonance; above this, came the hepatic dulness as usual, extending half a space higher than its normal limit. The swelling moved but very little, if at all, during deep inspiration. The pain came on in paroxysms, and, during them, the tumor became visibly more rounded, but there was no visible peristalsis. All the viscera seemed normal, and the temperature was normal. There was no ascites; no jaundice worth the name; no enlargement of the surface veins.

These, gentlemen, are the details of this case, wanting only that which no description can give—the something over and above the mere symptom which the eye takes in at once, and which is often all-important in making the diagonsis. However, I do not know that there was anything very striking in this man's appearance except that he was very pale, and looked proportionately ill. Will any one suggest a diagnosis?

Well, first of all, what may the symptoms indicate? A hard, somewhat nodulated lump between the umbilicus and the right ribs. In that region are the liver, gall-bladder, pylorus, head of the pancreas, the hepatic flexure of the colon, and, behind these, the suprarenal capsule and kidney, and some lumbar glands; and, lastly, mesentery and omentum may be displaced anywhere, and therefore must be mentioned. Bear in mind, too, that spinal curvature—lordosis, as this form is called—will sometimes so push forward the pancreas and aorta as to simulate an abdominal tumor or aneurism. To remember this, is to make your diagnosis so far as *it* is concerned; because it is, of course, easy to examine the length of the spine; and, when there is curvature of one part, there is nearly always curvature of another part compensatory; This patient has no spinal curvature.

Then to take the other possibles *seriatim.* Had he any liver affection? What liver diseases are liable to occur in a young man of twenty-seven? First, there is hydatid disease, which has these features: that the tumor is cystic; that, therefore, it gives the physical signs of fluid, viz., a thrill—in some cases a peculiar thrill—and the globular elastic tumor, generally speaking, bulging well into the right hypochondrium. It is a swelling comparatively free from pain. It is associated with little or no disturbance of the health. These are conditions, you will observe, that do not tally with those in this case. The tumor was not cystic, but very hard and unyielding. It was not even globular; nor did it occupy the region of a hepatic cyst; and instead of being unassociated with disturbed health, the man, as I have told you, was evidently very ill. In these latter points, however, it might agree with the symptoms of hepatic abscess or suppurating hydatid; but then, again, these would give the same cystic bulging of the liver if they gave any sign; and it was not present. Then some syphilitic disease of the liver—was it that? Syphilis leads to the formation of large hard masses in the liver, which produce much matting of the surrounding parts, and therefore occasionally give rise to a good deal of pain; so that it might have been something of that kind, even though there were no other traces or history of anything of the kind. And, lastly, was it any malignant growth in the liver? Well, that is so exceedingly uncommon as a primary disease in anybody; and in a young adult of twenty-seven still more so, that any opinion of that sort was mentioned only to be dismissed. So, then, of all the possible affections in the liver, we have only one left, viz., some gummatous inflammation. But I did not think it was a mass connected with the liver at all; first, because the tumor itself was distinctly resonant; and, secondly, because there was still more marked resonance between the lump and the ribs, above the margins of which came the normal hepatic dullness in due course. You occasionally find a large liver coming some way below the ribs, overlapped by or covered by some intestine; so that, though really enlarged, the abdominal portion is hidden; but I never saw a liver covered above by intestine and protruding below; and, if you think a minute, such a condition is hardly possible, considering the relation of the intestine to its mesentery. Such a condition is just possible with regard to the gall-bladder; that the coils of intestine might enfold the viscus, and become adherent round it, giving resonance above it, and some transmitted resonance over it; and the gall bladder is rather liable to set up inflammation round it, and fever. It becomes over-distended, and inflamed because over-distended. But I did not much think this was gall-bladder, because distension of the gall-bladder in young people is usually associated with some jaundice, and this was not; for though Mr. Malpas tells you the man was jaundiced, I think it would have been more correct to leave the earlier report, that the complexion was *sallow*, without the addition. He was sallow, no more. Then, too, the fundus of a distended gall-bladder is not often adherent to the surface to such an extent as to produce a lump so defined as this was. It is pyriform elastic swelling, to be felt only with care. Then, too, both liver and gall-bladder descend freely during inspiration; this tumor did not; though, of course that symptom is liable to be modified by adhesion, should this have occurred.

Then we may take the kidney and suprarenal capsule; and I may at once lay down this rule: that, unless very large or abnormally mobile, enlargements of these viscera do not produce any well-defined tumor so superficial as this was. A man may have twenty or thirty ounces of kidney in each loin, instead of four or five; and, unless there be any special renal symptoms, the tumors will very probably not be detected. This is because they are behind all the coils of intestine, and push these forward, so that the swelling is masked in this way. Renal tumors are indicated by a general fullness of the abdomen, or ill-defined resistance in either loin; and then, on careful manipulation, you will get fluctuation and so on. Now go and verify this for yourselves in the case of 19 in Philip ward. There is a man who has had symptoms of renal calculus on the right side. Go and examine his abdomen, and you will have learnt a lesson you will not forget. The same thing applies to the suprarenal capsule, though less generally, because growths in it of any size are apt to push forwards towards the median line, and may appear as definite tumors well defined, by pushing up the pancreas in front of them. As an outside chance, I put down tumor of the suprarenal capsule.

We have now left the pyloric region of stomach and colon, omentum and mesentery. The pancreas may be left out of the question, because I might say the same of it as Sir Walter Gull used to say here of the jejunum, "that it has no pathology." That is a fact, though, worth your thought. Sir W. Gull used, I say, to take the ileum, jejunum and duodenum, and enumerate the diseases of the first and the last, and find nothing to score against the jejunum. This is so; the jejunum is but rarely diseased primarily: and what is true of the jejunum is true of the pancreas, so far as we know at present; its diseases are not numerous. As between the remaining possibles, I said this: First, that the paroxysmal griping pain associated with vomiting that this patient suffered, and also the visible swelling of the abdomen which occurred at the seat of the tumor, together with the resonance over the tumor, were proof of some matting of the intestine, and so an impediment to its healthy, painless muscular action, or else of some positive obstruction. There was, however, no distension of the abdomen, and the bowels, though not regular, were not confined; so I thought there could be no obstruction of anything more than a temporary kind; and, under such circumstances, intussusception or any malignant disease of the colon were hardly likely. I therefore turned to the stomach, the patient being a young man, and a former hard drinker, and suggested that he had some ulcer there, which had, as do so many gastric ulcers, slowly perforated into the tissues outside; making its way downwards and to the right, instead of backwards; and that the inflammatory thickening so set up had caught the intestine, and led to the paroxysmal colic. That was my diagnosis; but, in making it, I had to do some violence to two sets of facts. The first was remarked upon by some of you, viz., that the man had no symptoms of gastric ulcer. To this I replied, that gastric ulcer gives so few symptoms in many cases, that the absence of them is not to be relied upon, especially in hospital patients, who take so little heed of their symptoms; and, moreover this man had "coffee-grounds" vomit, and passed black stools—very like some gastric hæmorrhage. Secondly, the swelling in the abdomen was so hard, that I was strongly disposed to think it must be a new growth of some kind; and I also remarked that you must bear in mind that growths in the stomach and intestine do occur at earlier ages than most other growths; and I mentioned the case of a woman which is on record, where carcinoma of the stomach caused death at twenty-one.

Closer than that, there was no ground for going; and, upon that diagnosis, and the prominent symptom being the one of paroxysmal colic, we gave him half a grain of opium three times a day, and kept him on milk diet, hoping that by this means the muscular coat of his intestine would go to sleep, and let the supposed ulcer or inflammation subside. Unfortunately, he was already, only we could not see it, in a hopeless condition; but let me continue the report of the case.

In the first two days he was sick, and had severe paroxysms of pain; still there was no visible peristalsis. The next two days he was quite comfortable, without sickness, the bowels acting with normal motions, and only one short paroxysm of pain. He was so comfortable, and complaining of feeling hungry, that I was somewhat thrown off my guard, and consented to his having a little fish. Whether from this increase of diet or not, I cannot say, but, on the evening of the day on which fish was taken, he had sudden and severe abdominal pain, which produced speedy collapse; and, notwithstanding a liberal administration of opium, wisely given by Mr. Horrocks, when I saw him the next day he had the sunken eye, the cold skin, the thready pulse, of a patient with acute peritonitis on the point of death. After this, he vomited everything given him, which was very little; for I believe the best treatment in peritonitis is to give no food at all, and plenty of opium; and he died about sixy hours after the onset of his last attack of pain.

The *post mortem* made the same afternoon discovered a cancerous stricture of the hepatic flexture of the colon. This had led to ulceration above it in the ascending colon, and a large fæcal abscess had formed in the right hypochondriac and lumbar region. Suppuration had extended from it to the viscera in the neighborhood, and so to the general peritoneum. We also found evidence of old peritonitis in the form of old adhesions; and these were associated with, and probably due to, old tabes mesenterica, or caseous and now calcareous disease of the mesenteric glands.

Now, remember my diagnosis was gastric ulcer, opening into the tissues outside the stomach, and so leading to the formation of inflammatory material, which had caught up the colon and impeded its action, but had not constricted its calibre to any great extent. Inflammatory products outside the stomach there were in plenty, but they originated not in the stomach, but in a stricture of much tightness in the colon.

How was it that we failed to diagnose this? Well, because proper or usual symptoms were absent. The symptoms of stricture of the colon are paroxysmal colic, visible peristalsis, constipation, and distention of the abdomen; and, of these, some are more significant than others. For instance, the paroxysmal pain is present in most intestinal inflammation and obstruction, whether in small or large intestine. The visible peristalsis may or may not be present; but, at any rate, in a tight stricture of the colon you would expect to find constipation and distention of the abdomen. If these be absent, I hardly know any condition which would enable you to make a diagnosis; and I do not see in this case, now that we have the *post mortem* to guide us, how we could have arrived at a perfectly correct opinion. If there be no distention, you will be justified in assuming that obstruction, if it exist, is very high up in the small intestine; or that it is a general contraction, such as I mentioned to you; or that it is not complete enough to hinder to any material extent the passage of the intestinal contents. I took the latter view in this case.

But the *post mortem* shows, I think, why the important symptoms were absent; and, in so doing, conveys a very important lesson that can be learnt in these cases. Remember that the stricture occurred at the hepatic flexure of the colon, and that above that, ulceration had occurred in the mucous membrane of the bowel, and a communication had thus formed between the intestinal canal and the loose cellular tissue outside in the loin. This had allowed of the escape of a quantity of the contents of the bowel into the large abscess we found, which was without doubt due to the ulceration of the intestine, and some relief to the distention of the bowel above the stricture.

Now, it is a well known fact of *post mortem* experience that strictures of the bowel are *never complete*. There is always a certain amount of channel left, usually enough to squeeze the index or little finger through. So that it is probable that the fatal obstruction occurs from some temporary condition of the canal, which converts the partial into a complete obstruction. The treatment of these cases quite confirms this. Many are the cases, even of those which eventually have proved themselves due to carcinoma of the bowel, where the temporary condition has been tided over and the complete obstruction relieved. The conditions which cause this appear to be either paralysis of the muscular coat of the bowel; or some alteration in the direction or irregularity in action of the muscular force, by which means the contents fail to be propelled through the narrow canal in front of them ; or some alteration in the contracted ring of bowel by which the existing canal becomes temporarily still more narrow. But whether one or all of these conditions hardly matters, because they all result more or less immediately from over-distention above the stricture. So that, in treating these cases, this is what you aim at, the unloading of the bowel above the stricture and keeping it as empty as possible. If you can do this, the symptoms disappear and the patient regains health, which of course will be lasting or not according as the stricture is carcinomatous or not, and according as the over-distention can be obviated in the future.

The most obvious way of accomplishing your object is, to those who have not seen the ill effects of their administration, to give purgatives, the *rationale* of such treatment being, no doubt, apparently to stimulate the muscular coat behind the obstruction and so to force the contents of the bowel through the stricture, but this is what does not happen ; purgatives seem to create somewhat analogous conditions to those occurring when a large theatre or church full of people is set on fire and all the occupants attempt to rush out at once. You know what happens. Half are crushed at the doors and the other half burnt inside. Convert that into terms of intestinal obstruction, and the result of purgatives is generally either to produce ulceration of the bowel above the stricture or to make the obstruction more complete than before.

The proper procedure first at hand is to reduce the contents of the intestine above and below the stricture to the smallest possible amount. Above, you are helped in this way by the vomiting which is usually present; and all that it will generally be necessary to do will be to be passive, and put no more in to replace that vomited. Your duty is to starve—yes, literally to starve—the patient. He may suck a little ice, and have a mouthful occasionally of the weakest broth ; and this rather to calm his mind than to sustain his strength. We need not mind about that. Most of these patients have a certain reserve in their blood and tissues to fall back upon ; and in bed strictly at rest they are in no danger of dying from a day or two of foodlessness. You will clear the intestine below the stricture by copious enemata frequently repeated. Then, as to drugs, the first and by far the most important one is opium. This controls and moderates the intestinal muscular action, and in so doing, without any other means, will often put a stop to the obstruction. So quickly does it accomplish this sometimes, that the opium pill would almost appear to act as a purgative, only that positions are changed now; purgatives constipate, sedatives open the bowel. If the symptoms are severe, give no more than opium and wait; but in the less urgent forms of obstruction, both belladonna and nux vomica are of use.

With regard to belladonna, Dr. Norman Kerr has published some remarkably successful cases of the subsidence of the symptoms of intestinal obstruction under one and two grain doses of the extract, given at frequent intervals. I have lately had a case under my own care where I pursued a similar plan, the patient taking twelve grains of the extract in about thirty-six hours; but, unfortunately, the result was not successful; peritonitis supervened, and death took place as colotomy was being performed. I do not wish to imply that this treatment is valueless. I think now that the case was perhaps of too long standing to allow of any hope of success except by operation; and would suggest that the belladonna should be exhibited in the earlier days of the obstruction if it is to succeed. We certainly lost valuable time, which might have allowed of successful colotomy, while pursuing the belladonna treatment.

I see no reason why ergot and digitalis should not be of use also; though they are less generally applied to. All such drugs, acting as tonics to the muscular coat of the bowel, induce a more persistent and forcible *vis à tergo*, replacing an irregular and inefficient muscular action. These, with warmth to the abdominal parietes, will be successful if any measures are of avail, and it will not often be necessary to do more by way of drugs. Some advise the administration of oil and other fluids to liquefy the intestinal contents; this is quite unnecessary, and even harmful, by adding to the quantity of liquid above the stricture, for it is a rule, to which I have seen no exception on the *post mortem* table, that *the intestine above the stricture is distended by fluid fæces, not by lumpy matter.*

Well, then, should all these measures fail, and you have to do with stricture of the colon, and have only to consider the question of the relief of the stricture apart from its nature and other complications, then the proper thing to do, no doubt, is to open the bowel above the stricture by some surgical operation. Now, in deciding upon such a thing as this, it is perhaps more common to look upon the operation as a way out of a difficulty by avoiding it; as a means to secure a permanent opening above the stricture — an artificial anus in fact. But — and this is the point I wish to insist upon, for here it is that one of the points of our case comes out — in many cases you will find that after the colotomy the bowels act and continue to do so by the ordinary channel. You relieve the over-distension by the operation, and the obstruction ceases. So that the operation is remedial in such. You do, in fact, by operation what you have previously attempted by drugs, and do not merely use a makeshift.

A very interesting case of this sort has been published in the *Guy's Hospital Reports* by Mr. Hilton. A medical man suffered for some time from abdominal pain and constipation, and eventually the obstruction became complete. Mr. Hilton saw him after twenty-eight days, and agreeing with the other medical men in attendance that the seat of disease was in the rectum or sigmoid flexure, opened the colon in the left loin. Four days afterwards, evacuations began to pass *per anum*, and from that time this continued to be the case; and the opening in the loin gradually healed. Eleven weeks after the first operation, all the old symptoms returned, and it became necessary to reopen the colon, when again fæces passed naturally *per anum;* and again — though this time vigorous efforts were made to dilate the artificial opening, the incision healed at the end of eleven weeks. The patient went on for some weeks, when it became necessary to open the colon a third time. But by this time ulceration had occurred in the colon above the stricture, and a large abscess had formed outside the bowel, opening the hip-joint, and from which he ultimately died exhausted. He lived a year almost to a day from the time he was first taken ill, and eleven months after the first operation.

In our case, you will remember, there was no distension of the bowel, and nothing that could be called constipation, and I attribute their absence to a similar reason to that which existed in the case just narrated; viz., to the presence of a safety valve above the stricture, the only difference being that in the one case it was made by the surgeon, in the other by the spontaneous morbid process of ulceration; and in our case, instead of being remedial it was a case of "out of the frying-pan into the fire." I can only suppose, however, that the ulceration which we found in the ascending colon had allowed the escape of fæcal matter into the cellular tissue of the right loin, and in this way had in some measure relieved the distension which must otherwise, almost of necessity, have ensued above the stricture, and so the bowel was allowed to act.

You may perhaps think that some less hazardous means of relieving the distension than that of colotomy might be adopted, and another operation has been practiced with that end in view, viz., paracentesis. The distension is due partly to gas and partly to fluid fæces; and it has been thought that by withdrawing the former the severity of the case might be relieved. One of the distended coils has therefore been tapped by a very fine trocar and cannula. But there can be no doubt that this is an exceedingly dangerous thing to do; and I do not, from what I have seen and others have told, feel in the least inclined to recommend it to your notice. The danger is this: that the distension is, in the majority of cases, but little relieved — that alone is an objection fatal to its adoption — and the bowel remaining full and its walls tightly stretched, fæcal matter, which you remember I told you is *always* liquid, leaks out into the peritoneum after the withdrawal of the cannula from even the smallest puncture. I have myself seen this operation performed, and fæcal matter came out at once by the cannula, no relief followed, and the patient died not long afterwards of acute peritonitis. So do anything rather than this. You are taught, and quite correctly so, that small wounds of the intestine are comparatively dangerless, because the mucous membrane becomes everted and so closes the aperture; but this only applies to a contracted intestine; we are dealing with an overfull one. All the coats are in such a case distended probably to their utmost, the rugæ obliterated, and there is nothing to evert; and the smallest hole, under such circumstances, becomes a vent, and a vent, however small, in such a position, is fatal. Of course, all these points are beside the question in our case, because there was no distension of the abdomen and no constipation, so that we had nothing to consider but the treatment by drugs.

One other fact in our case must be alluded to, if for no other reason, because you will not find much about it in your books. We found evidence of old peritonitis, and the glands in the mesentery were caseous or calcareous, and there had evidently been a so-called tabes mesenterica of former date, from which the patient had recovered. That condition is of sufficient interest in itself to devote a lecture to; but the point in this case is, that such a state of things must of necessity somewhat modify the distension which we should expect as the result of obstruction, and might in some cases prevent it entirely. It may, in conjunction with the other previously mentioned conditions, have so acted here. It is quite obvious that if the intestinal coils are matted together and to the surrounding parts, and the mesentery itself be shortened and thickened, there is less chance at any rate of the usual uniform distension, and it may be, as I say, that none will be present.

This patient has been rich in lessons, in that the poor fellow is dead; but there is another patient now in Bright ward, whom you should go and examine — though do so tenderly — to compare with this. The case presents a similar amount of speculative interest as that I have just detailed did, concerning the exact nature and seat of the disease. I cannot now do justice to the very careful report of the case by Mr. Maylard; but, shortly stated, these are some of the features of it. She is thirty-five years of age, and was admitted into Bright ward under my care on March 1st, 1878. She is a married woman, of phthisical family. She has had six miscarriages and four living and said to be healthy children. Her last confinement was thirteen months ago. She appears to have suffered many things of many physicians, or rather surgeons, and has been an inmate of at least four of the metropolitan hospitals for typhus, rheumatic fever, erysipelas, etc. Seven months before her admission, she noticed pain in the right umbilical and epigastric region whilst mangling. She vomited, and the pain was persistent. She tacked about between various dispensaries for two months or more; and a swelling was noticed in the abdomen by Dr. Stocker. A fortnight before she came here, she was seized with severe abdominal pain; vomiting, which all along had been occasional, had been much more severe; and for three days she had been unable to retain anything on her stomach. The bowels had been very confined, rarely acting under intervals of from four to twelve days. She looked pale and ill; and on examining the abdomen we found a tumor to the right of the umbilicus, a little lower than in the former case, but like it giving some resonance on percussion, and there was still more resonance between it and the margin of the ribs. It was ill defined at its margin, very tender to manipulation, and not displaced by deep inspiration. The abdomen was not distended. The morning temperature was over 100° F.

The difficulties in diagnosis here are similar to those in the man. The tumor has not in this case any of the hardness present in the other, and its position is somewhat lower; and for that reason, the ascending colon and cæcum must be added as possible seats of disease; and conning over the case, I look upon it as one of localized peritonitis, the cause of which is not evident. Possibly it is due to some ulceration of the colon or displaced cæcal appendix, possibly to disease of the gall bladder. We gave her half a grain of opium and a quarter of a grain of extract of belladonna in a pill three times a day, with hydrocyanic acid and bismuth, milk, lime- and soda-water; and, under that treatment, she had no return of sickness, though before it had been constant. Since that time, the chief additional symptoms have been that the tumor has shifted up a little, so that if you examine it now you might think it to be attached to the liver; that she has had throughout very considerable abdominal distension; that the bowels have been decidedly confined; and that she has had decidedly more distension and abdominal pain on a fuller diet than on a restricted one. There is, moreover, a history that, on April 3d, she passed a stone about as big as the top of one's thumb in her motion. The nurse stupidly threw it away, so we can only conjecture that it may have been a gall-stone. If this be so, a pretty good case would be made out, for gall-stones encysted in the gall-bladder; ulceration of the gall-bladder; adhesion by inflammation to the part around; ulceration into colon or some part of the intestine low down; and some amount of constriction of the bowel as a result of the inflammation. But this is all very conjectural, because she has repeatedly passed scybala, and the supposed gall-stone may have been no more than a hard fæcal nodule; and our diagnosis practically rests where it did at first at chronic peritonitis catching up the colon and producing partial obstruction. The treatment has been such as I described, and she has latterly had copious enemata by the rectum. The prognosis in the case is somewhat gloomy; she is getting tired of the hospital, and will go out before long. She will then pay no attention to her diet or bowels, and will in that case soon get complete obstruction; though I believe if she could be kept under observation and guidance she might get well.—*British Medical Journal.*

